# A cross-sectional survey of 5-year-old children with non-syndromic unilateral cleft lip and palate: the Cleft Care UK study. Part 1: background and methodology

**DOI:** 10.1111/ocr.12104

**Published:** 2015-11-16

**Authors:** M Persson, J R Sandy, A Waylen, A K Wills, R Al-Ghatam, A J Ireland, A J Hall, W Hollingworth, T Jones, T J Peters, R Preston, D Sell, J Smallridge, H Worthington, A R Ness

**Affiliations:** Centre for Appearance Research, University of the West of EnglandBristol, UK; School of Oral and Dental Sciences, University of BristolBristol, UK; Dental & Maxillofacial Centre, Royal Medical ServicesWest Riffa, Kingdom of Bahrain; Children’s Hearing Centre, University Hospitals Bristol NHS Foundation TrustBristol, UK; Centre for Child & Adolescent Health, School of Social & Community Medicine, University of BristolBristol, UK; School of Social & Community Medicine, University of BristolBristol, UK; Musgrove Park HospitalTaunton, UK; School of Clinical Sciences, University of BristolBristol, UK; Cleft Lip and Palate AssociationLondon, UK; Speech and Language Therapy Department and Centre for Outcomes and Experience Research in Children’s Health, Illness and Disability (ORCHID), Great Ormond Street Hospital NHS Foundation TrustLondon, UK; South Thames’ Cleft Unit, Guy’s and St Thomas HospitalLondon, UK; Cleft Net East Cleft Network, Addenbrooke’s HospitalCambridge, UK; School of Dentistry, University of ManchesterManchester, UK; National Institute for Health Research (NIHR) Biomedical Research Unit in Nutrition, Diet and Lifestyle at the University Hospitals Bristol NHS Foundation Trust, University of BristolBristol, UK

**Keywords:** cleft lip, cleft palate, cross-sectional studies

## Abstract

**Objectives:**

We describe the methodology for a major study investigating the impact of reconfigured cleft care in the United Kingdom (UK) 15 years after an initial survey, detailed in the Clinical Standards Advisory Group (CSAG) report in 1998, had informed government recommendations on centralization.

**Setting and Sample Population:**

This is a UK multicentre cross-sectional study of 5-year-olds born with non-syndromic unilateral cleft lip and palate. Children born between 1 April 2005 and 31 March 2007 were seen in cleft centre audit clinics.

**Materials and Methods:**

Consent was obtained for the collection of routine clinical measures (speech recordings, hearing, photographs, models, oral health, psychosocial factors) and anthropometric measures (height, weight, head circumference). The methodology for each clinical measure followed those of the earlier survey as closely as possible.

**Results:**

We identified 359 eligible children and recruited 268 (74.7%) to the study. Eleven separate records for each child were collected at the audit clinics. In total, 2666 (90.4%) were collected from a potential 2948 records. The response rates for the self-reported questionnaires, completed at home, were 52.6% for the Health and Lifestyle Questionnaire and 52.2% for the Satisfaction with Service Questionnaire.

**Conclusions:**

Response rates and measures were similar to those achieved in the previous survey. There are practical, administrative and methodological challenges in repeating cross-sectional surveys 15 years apart and producing comparable data.

## Introduction

The organization of care for children born with a cleft of the lip and/or palate in the United Kingdom (UK) underwent significant change over the last 15 years as the Clinical Standards Advisory Group (CSAG) report was published in 1998 1 and the onset of the CCUK study. The outcomes of the CSAG study were widely reported, and there have been considerable operational and service reconfigurations in this area of UK healthcare since those reports 2–6. The 57 centres operating on children born with some form of oro-facial clefting in the UK in 1998 have been reduced to 11 centres or managed clinical networks. The implementation period has been prolonged, but all cleft services are now in an ongoing process of centralization. The impact of these changes on care and outcome is unclear 7.

The CSAG was set up by the UK Health Ministers in 1991 as an independent source of expert advice on access to availability of selected National Health Services (NHS) specialized services. A number of areas were examined including childhood leukaemia, schizophrenia and women in normal labour. For cleft lip and palate, the CSAG committee commissioned a research team to undertake studies of non-syndromic cases of unilateral cleft lip and palate (UCLP) in children aged five and twelve years throughout the UK. A cross-sectional survey of the process of care assessed key outcomes including speech, hearing, dento-alveolar and skeletal relations, bone grafting, facial appearance and patient/parent satisfaction. The CSAG survey team identified 326 5-year-olds born with UCLP in the UK over a 2-year period, and outcome records were collected for 239 of these children. This represented 73% of those eligible. A full account of the methodology is presented elsewhere 1. The findings of the report were far-reaching and accepted for the following reasons:

1. The evidence in the CSAG report was compelling and arose from a detailed and meticulous observational study.
2. The recommendations of the CSAG report were supported by all members of the CSAG committee including those whose clinical practice was likely to be directly affected by implementation.
3. The recommendations were accepted by government and supported by the relevant professional organizations.
4. The proposed changes were in line with evidence from centres abroad, which were generally agreed to provide excellent services.
5. Virtually all cleft care in the UK is provided within the NHS.
6. Most importantly, there was, and still is, an active and well-informed patients/parents/professionals group (Cleft Lip and Palate Association, CLAPA) that accepted in principle the recommendations of the CSAG report.

The key recommendation, unanimously supported by the CSAG committee, was that the number of centres offering cleft services in the UK should be reduced from 57 to around 8–15. Inevitably, not all regions could implement these changes at the same time but there were opportunities for the individual centralization processes to learn from each other and to request direction from the Department of Health.

In the years after the CSAG report, there have been preliminary studies in secondary alveolar bone grafting and dento-alveolar relations that have reported improvements in outcomes 8,9. These evaluations took place in the early stages of centralization and were either regional (rather than national) or not comprehensive in scope. Given these shortcomings, a national examination of the impact of these changes to cleft services some 15 years after the centralization process was both timely and relevant. In this study, we describe design issues, the conduct of the fieldwork and the data collection methods used in the study. We have included detailed descriptions of the coding and analysis of these data in the later studies in this supplement. We focus the results and discussion of this study on the comparability of measures and response rates with the previous survey.

## Subjects and methods

The original cross-sectional CSAG study did not require research ethics committee approval as it was considered to be an audit. However, because of the changes in ethics procedures and a wish to incorporate collection of research data into the present survey, ethical approval was obtained (REC reference number: 10/H0107/33, South West 5 REC). Approval included consent to link medical and other records in a number of areas (such as education) and for additional measures (height, weight, head circumference) and questions (psychosocial, health and lifestyle and economic) to be collected. There was also approval to approach families for further research in future.

### Study design

The original CSAG survey published in 1998 1 was cross-sectional and attempted to locate and study all 5-year-old children born between 1 April 1989 and 31 March 1991 with non-syndromic UCLP. To compare a similar group post-centralization, we conducted a further cross-sectional study of 5-year-olds born with UCLP (known as Cleft Care UK) treated within a centralized or centralizing service. We adopted a protocol that was as similar as possible (although extended in places to include additional items) to the original study. It was not felt necessary to survey all children in the UK born with all expressions of clefting as several multicentre comparisons have provided evidence that care for, and outcomes in, UCLP cases are representative of the quality of care and outcomes in a centre 3. The original CSAG survey also included 12-year-olds. At the time of the current study, however, this age group would not all have been cared for in a centralized service so they were not resurveyed.

### Participant eligibility

**Figure 1:**
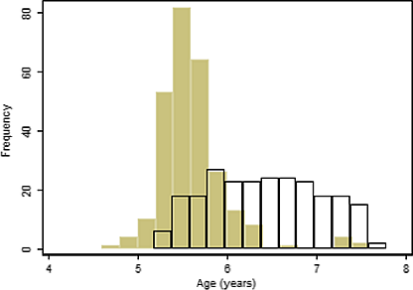
Histogram of the age distribution of the CCUK (filled bars) and CSAG children at presentation to the data collection clinics.

We collected records of 5-year-old children from cleft centres in the UK born during a 2-year period. The original inclusion criteria comprised the following:

- Five-year-old children born with non-syndromic complete unilateral cleft of the lip and palate, including any with soft tissue Simonart’s bands of less than 5 mm.
- Children born between 1 April 2005 and 31 March 2007.
- The child was aged between 5 years 3 months and 5 years 9 months. If a child failed to attend the initial scheduled research audit clinic, they were invited to attend a subsequent audit clinic up until the age of 6 years and 5 months. Some children were seen at younger and older ages than originally stipulated (Fig.1); we decided to include these children and to examine and adjust for age in analyses where appropriate.

Exclusion criteria comprised the following:

- Children born with UCLP whose developmental delay was sufficient to prevent them from cooperating with procedures (such as speech recordings) that were needed for data collection.
- Refusal to participate in the study by either parents or children.

We did not exclude cases until they had been discussed, on a case-by-case basis, with the cleft centre and the research team during the scheduled audit clinic. Cleft centres were asked to provide clinical photographs of those children born with UCLP and excluded from the study because they had soft tissue Simonart’s bands of more than 5 mm in width.

### Study clinics

Post-centralization, regular audit clinics were set up by most cleft centres, and defined age groups (including children around the age of 5 years) are now routinely reviewed. Measures of outcomes for appearance, dental arch relationship, speech and hearing (function), oral health and psychosocial adaptation are collected at these audit clinics. To support the research study, cleft centres agreed to organize designated audit clinics and invite eligible 5-year-olds within the agreed age range. These clinics collected additional information to standard audit clinics (such as head circumference); hereafter, we refer to these clinics as audit clinics. The research team (comprising psychologists and dental academics) liaised and worked with an identified key member of each cleft centre to arrange dates and details of audit clinics and to collect data. Eligible families were sent written information about the study from the cleft centre together with their appointment details for the audit clinic. On the day of the audit clinic, the parent(s) and their child were asked whether they would participate in the study and, if they agreed, consent was obtained from the parents and assent obtained from the child. The research team sought consent and assent for the majority of children, but if the researchers were unable to attend the audit clinic, the clinical team at the centre sought these agreements. If the parent refused to participate in the study, the data obtained during the clinical examinations were stored in the child’s medical files and not accessed by researchers. If the parents and child failed to attend their initial audit clinic, further invitations were sent until no further audit clinics were organized and therefore available. Audiology and speech assessments and recordings were carried out by the centre specialist team or by local specialist staff (i.e. audiologists and speech and language therapists). Dental examination and assessment were carried out by British Association for the Study of Community Dentistry (BASCD)-calibrated 10 paediatric dentists, and psychological assessments were completed by a psychologist. In those centres that did not have access to a paediatric dentist or psychologist, the research team conducted the assessment. Standardized photographic views (extra-oral and intraoral) were taken by either the medical photographer or the orthodontist at the cleft centre. Impressions for dental study models were taken by the orthodontists, and they also completed details of the child’s orthodontic history. The surgical forms were completed by surgeons using information from the medical notes. The surgeons also assessed the appearance of the lip and nose. Parents/guardians were given a number of questionnaires to be completed in the audit clinic and placed in a questionnaire box provided by the research team. Alternatively, they could mail them back in a free-post labelled envelope provided to them. The parent/guardian was also asked to complete the Health and Lifestyle Questionnaire and the Satisfaction Questionnaire in their home environment, to be returned to the researchers in a free-post labelled envelope. If questionnaires were not returned within 7–14 days, the research teams posted one reminder with a further copy of the questionnaires together with a free-post labelled envelope.

### Surgical treatment

The type of primary lip repair, surgical complications, type of palate repair and whether antibiotics were used at the time of lip and palate closure were recorded from medical notes. The surgeons examined the child to assess whether any oral fistulae were present and whether there were any functional problems such as nasal regurgitation of liquids and food.

The surgeon also documented their subjective rating of the surgical outcome using a four-point Likert scale ranging from poor to very good on each of the following: the scar quality of the lip, Cupid’s bow, lip length, frontal view and inferior view of appearance/symmetry of the nose. The vermillion border was assessed on three parameters: notching and/or deficiency, was it balanced (i.e. equal on both sides), or was it or too full or bulging. The functionality of the lip was assessed when smiling (symmetrical/equal length or asymmetrical/shortened) and pouting (symmetrical or asymmetrical).

### Assessment of dental arch relationship

Alginate impressions of the child’s teeth were taken together with a wax squash-bite in centric occlusion and a record of the overjet. The impressions were placed in labelled plastic bags and transported to an orthodontic laboratory by the research team in a ‘cool bag’. For consistency, a single laboratory technician handled all of the impressions and constructed all of the models. The impressions were cast in white plaster and study models constructed in a standardized format with the participants ID number inscribed in the base. Some cleft centres provided existing study models, which were then duplicated in the laboratory and the original returned to the centre. If impressions could not be obtained, then intraoral photographs of the teeth in occlusion were taken. These intraoral photographs included a frontal view, right and left lateral views and, if possible, a palatal view. The interarch relationships of the 5-year-olds’ study models were used to indicate the effects of surgery on dental arch relationships, and this was assessed with the well-established 5-Year-Olds’ Index 11.

### Facial aesthetics

A two-dimensional assessment of the child’s face was made from profile and frontal photographs that were taken using published guidelines 12,13. Photographs were taken with a standardized camera set-up (Camera Nikon D3s or other equivalent camera, a 105-mm macro lens and lighting equipment for the camera). The child was asked to sit on a chair positioned 0.5 m in front of a standardized black or white non-reflective wall mounted background. If necessary, the child’s hair was arranged to show the entire ear.

The following views were taken for each child with the lens length fixed to give standardized magnification (the numbers in brackets are the magnification settings on the camera): left lateral face (1:8), right lateral face (1:8), ¾ left lateral face (1:8), ¾ right lateral face (1:8), facial (1:8), facial smiling (1:4), whistling (1:4), worm’s eye view (1:4), lip and nose (1:3).

The images were anonymized and cropped to allow unbiased assessment of only the nose and lip area. They were rated independently by a panel of assessors to determine the appearance of the lip, nose and profile of 5-year-olds. To evaluate the parents’ perception of their child’s appearance, the 20-item Satisfaction with Appearance Scale was used 14–16.

### Oral health

The child’s dental history was derived from parental accounts and checked with hospital notes where available. Information included identification of the usual care provider (general dental practitioner or specialist paediatric dentist), the use of neonatal appliances and past dental treatment. A clinical record was made of the buccal occlusion, oral cleanliness and the number of decayed, missing and filled teeth (dmft). The presence/absence of fistulae was also noted but only if a surgical assessment (where fistulae were also recorded) was not conducted on the day of the audit.

### Audiology

The history of audiological and otological interventions was derived from the medical notes and through questioning the parents. This history included the management of middle ear effusion through watchful waiting, insertion of grommets, the use of hearing aids (past and current) and other medical otological procedures. The audiological/otological assessment comprised a full audiogram which tested air conduction (AC) and bone conduction (BC) as appropriate to assess hearing thresholds. The management, type and degree of hearing loss were recorded on the audit assessment day with middle ear function assessed by tympanometry and otoscopy.

### Somatic growth

An assessment of growth was made by measuring the child’s height, weight and head circumference. The height was measured in centimetres to one decimal place using the Leicester Height Measure Scale (First Aid Warehouse, 17 Chesford Grange, Woolston, Warrington, WA1 4RQ, UK). The child was asked to remove their shoes and then positioned on the height measure with their feet flat, the underside of their heels in contact with the ground and the backboard of the measuring device. The heels were placed together, so that the medial malleoli were touching (unless the child had ‘knock knees’). The child was asked to stand straight with the shoulders relaxed and sloping forward in a natural position. The hands and arms were loose and relaxed with the palms facing medially. The head was positioned with the Frankfort plane (the line between the lower orbit of the eye and the upper margin of the external auditory meatus) parallel to the floor. Weight was measured in kilograms to one decimal place on a calibrated Seca weight scale (model 899; Our Weigh Ltd, 10 Fore Street, St Mary Church, Torquay, Devon, TQ1 4NE, UK). The child (lightly clothed and no shoes) was asked to step on to the middle of the scale.

Head circumference was measured in centimetres to one decimal place, with the child sitting comfortably and relaxed with the Frankfort plane parallel to the floor. The measurement was taken with a reusable Lasso-O tape from Harlow Printing Ltd (Maxwell Street, South Shields, Tyne & Wear, NE33 4PU, UK) with the tape being taut but not tight. The head was measured at the widest horizontal circumference above the eyebrows and ears.

### Speech methods

The assessment of speech included a therapy history questionnaire which captured information about current and past experience of speech and language therapy services. Details were gathered regarding waiting times for intervention, amount of contact time, location, focus of therapy, therapists’ views of contributing factors to outcomes and assessment of residual needs for speech and language therapy. Factors the speech and language therapist judged had contributed to the outcome were identified. Data on suspected and confirmed velopharyngeal insufficiency and history of velopharyngeal surgery were also gathered. The history was taken in the audit clinic by the speech and language therapist with information from parents, the medical notes and local SLT notes. If needed, the therapist subsequently sought further information from local services. An estimate of residual needs for speech and language therapy was made. This estimate was based on the clinical assessment made by the specialist therapist at the time of the recording. The speech outcome measures used the Cleft Audit Protocol for Speech-Augmented (CAPS-A) tool 17 developed in the UK for audit purposes in response to the CSAG report. For the assessment, speech audio–video recordings were collected using the equipment, procedures and speech sample as described in detail elsewhere 18. All recordings were made by one of the centre-based SLTs, who had been trained in the CAPS-A. Recordings were made in a quiet room with the child facing natural light if possible. A microphone was placed on a stand, 23–30 cm away from the child, at the level of their mouth and to one side. The face and upper neck were framed in the picture. The speech sample picture material was presented beside the camera at the child’s eye level. Following data collection, the SLT checked, using the headphones, that a high-quality sample had been recorded.

The speech sample comprised the following:

1. A sample of 2 min of conversation, which was encouraged with open-ended questions through a progression of enquiries on a particular topic.
2. Counting from one to twenty and from 60 to 70.
3. Saying (not singing) a nursery rhyme such as Jack and Jill.
4. Repetition of each of the 16 sentences after the therapist using the Great Ormond Street Speech Assessment picture book.

Assessment included nasality (hypernasality, hyponasality), nasal airflow (nasal emission, nasal turbulence), cleft speech characteristics, non-cleft speech immaturities and intelligibility/distinctiveness. This is described in detail in the speech study of this series.

### Psychosocial factors

These data were collected using a standardized questionnaire of 18 items that was originally developed by the Royal College of Surgeons of England Steering group 19 for the first CSAG study. Since the implementation of centralization, this questionnaire has been modified by the Psychology Specialist Interest Group (SIG). The final version of questions used in the study to assess parental/guardian perceptions of the impact of the cleft on their child was agreed by the Psychology SIG and the researchers before data collection began. The Strength and Difficulties Questionnaire with 35 items was used 20.

### Health and lifestyle

Three questionnaires were used to collect basic demographic data on the child’s temperament, potential determinants or modifiers of psychological adjustment, and family costs of parental preferences about the child’s cleft care. The basic socio-demographic section included questions on ethnicity, parental age at the birth of the child with a cleft, the highest level of parental education, most recent parental occupation, number of people living in the home and relationship to the child as well as details of other family members with cleft lip/palate and their relationship to the child born with cleft lip and or palate. In the Health and Lifestyle Questionnaire pack (140 items), established and validated questionnaires were used to assess the individual characteristics of both the parent and child. These included the Emotion, Activity and Sociability Scale 21, Parent–Child Relationships 22, Vulnerability and Over-protection 23, the Warwick–Edinburgh Mental Well-being Scale 23,24, the Life Orientation Test – revised 25 and the 10-item Perceived Stress Scale 26. The costs and discrete choices questionnaire measured personal and indirect costs of the child’s care incurred by the parent/guardian and was either sent out to families who attended early audit clinics or included in the packs handed out at the audit clinic for the later recruits to the study. The questionnaire was developed by the research team. A discrete choice experiment (DCE), using best–worst scaling 27, was developed to estimate the relative value of different attributes of centralized cleft care services 28. The first stage of the DCE involved the identification of attributes through a review of the literature and semi-structured qualitative interviews with parents/guardians of children born with UCLP. The second stage entailed the assignment of levels to the attributes identified, which was explored with parents/guardians during the semi-structured interviews. The final stage will involve asking parents/guardians to choose between two or more hypothetical service configurations that have been created to contain different combinations of attribute levels. Respondents’ choices are assumed to reflect the underlying value (utility) they place on service attributes 29.

### Satisfaction with service questionnaire

An additional questionnaire was used to assess parental satisfaction with the cleft centre service delivery.

## Results

### Overall recruitment

The cleft centres identified 359 eligible children born in the period 1 April 2005 to 31 March 2007, and this figure was corroborated through the national database CRANE (https://www.crane-database.org.uk/). Eighty-five families failed to attend the initial as well as the subsequent research audit clinics they were offered and were not part of the study. Six families declined to participate (2% of the 274 approached). Reasons included not wanting to participate, lack of time to complete the additional questionnaires or difficulties in re-arranging audit clinics to suit them. We collected data between 12 January 2011 and 12 December 2012 but continued to request missing data from the cleft centres until 30 April 2013. The study was officially closed on the 15 May 2013 when we had recruited 268 (87 females, 181 males) of the 359 eligible children 5-year-olds born with UCLP (74.7%). Of these, 18% had a Simonart’s band.

### Age of children

The cleft centres aimed to bring children to the audit clinics when they were aged between 5 years 3 months and 5 years 9 months. This was not possible for all children as it required cleft centres to schedule special audit clinics that would take place when the children were at the required age. We agreed that they should not run additional audit clinics to avoid increasing the burden on staff and families. As a result, 20 children (7%) attended the audit clinics before the age of 5 years and 3 months. One hundred and eighty-three children (68%) attended the audit clinics within the optimal time span, but others missed their appointment and had to be rescheduled. Thus, 65 children (24%) were older than 5 years 9 months when they attended an audit clinic. The mean age in the study was 5 years and 7 months (range 4 years and 6 months to 7 years and 6 months).

### Availability of clinic data

Table1 outlines the assessments made and the number of children who took part in each study. The research teams attended 86 audit clinics across the UK. At the audit clinics, it was possible to obtain 11 assessment records for each child. As there were 268 participants, a total of 2948 assessment records were, in theory, obtainable. For a number of reasons (e.g. the child would not cooperate or had missed the assessment), it was not possible to obtain all records. In the event, 2666 data assessment records were at least partially complete (90.4%).

**Table 1:** Number of children with data on each assessment in the Cleft Care UK study and comparative information from the CSAG study 1

Specialty	Obtained from 5-year-olds who attended the Cleft Care UK study	Obtained from 5-year-olds who were part of the CSAG study 1998
Audiology	n	n
Speech and language forms	227	200
Speech recordings	261	268
Anthropometry	248	238
Facial aesthetics	242	–
Oral health	252	200
Dental study models	264	239
Orthodontic form	198	223
Surgical details	263	239
Psychology questionnaire	243	297
Strength and difficulties questionnaire	253	220
Health and lifestyles (postal) questionnaire	215	–
Satisfaction with Service	141	–

### Self-completion questionnaire response rate

Two hundred and forty-six questionnaires (Health and Lifestyle and Satisfaction with Service) were given to families in the audit clinic for completion at home and postal return to the research team. Twelve families declined to answer the questionnaires because of either language difficulties or time constraints. Ten families did not receive information or received the questionnaires during the audit clinic and as a result did not participate in this part of the study. The response rate (those who returned the questionnaire having completed at least a part of it) was 52.6% (n = 141) for the Health and Lifestyle Questionnaire and 52.2% (n = 140) for the Satisfaction with Service Questionnaire.

### Comparison with the previous CSAG survey

The 74.7% recruitment rate in CCUK is marginally higher than the 73% recruitment rate achieved in the original 1998 CSAG survey. Table1 describes the response rates for CCUK and CSAG; the figures were broadly similar.

Table2 shows the age, sex, ethnic and socio-economic characteristics of the children in the CCUK and CSAG surveys where data were available. The CCUK children were assessed closer to the target age of 5 years – Fig.1 shows that the CSAG children were older (difference in medians: +0.9 years) and had a wider spread of ages. The sex and socio-economic distributions (Townsend index of deprivation) of the two cohorts were similar.

**Table 2:** Description of the study sample in CCUK and the 5-year-old group from the original CSAG study 1

	CCUK		CSAG		*p*-Value‡
	N	Median (IQR) unless stated	N	Median (IQR) unless stated	
Male (n, %)	268	181 (67.5%)	239	159 (66.5%)	0.80
Age (years)	268	5.5 (5.4–5.7)	239	6.4 (5.9–6.9)	<0.001
White ethnicity (n, %)	121	111 (91.7%)	–	–	
Deprivation score (ranking out of 100)*	210	21.1 (11.7, 35.7)	94†	20.2 (11.3, 32.4)	0.58

* English Index of Multiple Deprivation based on postal codes: 2007 for CCUK and 2004 for CSAG (http://geoconvert.mimas.ac.uk/help/faq.htm#walkthrough). Geocoding began in 2004 so this is the earliest comparable index available for CSAG. Measurement range = 0/100 in percentiles where lower scores indicate the most deprived postal areas.

† Postcodes were only partially recorded.

‡ *z*-Test for proportions and Wilcoxon rank sum test of medians.

## Discussion

We set out to collect data from all 5-year-olds born with non-syndromic UCLP in the UK over a 2-year period using comparable methods to the previous 1998 CSAG survey 1. This included all those with Simonart’s bands, which comprised approximately 18% of the sample. Five-year-old children were examined as they were likely to have received all their care within a centralized service. We recruited 268 participants and obtained data on most clinical outcomes for over 90% of those enrolled.

### Comparison of response rates between surveys

Although the response rates for these two surveys were similar, we had expected that post-centralization recruitment to CCUK would be higher. We thought that we would find it easier to recruit from fewer centres that had dedicated cleft teams working to defined job plans and running regular audit clinics. However, we did manage to recruit individuals closer to the target age in CCUK vs. the CSAG survey. The data in the CSAG 1998 study were mainly collected by the research team, whereas in Cleft Care UK, more of the data collection was undertaken by the cleft centres. As CSAG was approved as an audit project, information was collected from the clinical records of children even if they did not attend. In the CCUK study, however, information was collected only for those children who attended the audit clinics and where their parents gave informed consent. In the CSAG study, parents completed the Satisfaction with Service Questionnaire when they attended the audit clinic, whereas in CCUK, questionnaires were completed at home and returned by post. Centralization does require families to travel further for treatment and to attend audit clinics. This may in part have explained why more people did not attend the centralized audit clinics.

### Audit vs. research

The Cleft Care UK research team supported the local cleft centres with obtaining consent and assent and in data collection. There were delays in obtaining research approval for this study that we have highlighted elsewhere 30 and reduced response rates as a result of the need to obtain consent. However, obtaining research permissions has had advantages. It has made it possible to approach participants with further research questions at a later date. For example, the DCE was not included in the original CCUK protocol. It has also allowed us to develop research capability and capacity within the centralized service that will be available to support future research projects 31.

### The use of different clinical outcomes

Speech is recognized as an important functional outcome measure of cleft care. Enabling children with cleft palate to have normal speech by 5 years of age is a shared goal for both the family and the cleft team as it is important both socially and educationally. Britton et al. 32, in fact, argue that speech outcomes represent a cleft team’s multidisciplinary outcome, encompassing timely and effective primary surgery, well-coordinated follow-up, proactive hearing management, effective speech and language therapy, prompt and appropriate revision surgery where necessary, as well as recognizing a families’ commitment to care.

Facial growth is a key outcome. Study models of 5-year-old dentition can be used as a surrogate for likely future growth 11. We were therefore surprised that the Cleft Care UK study collected fewer study models (77.2% vs. 93.3% in CSAG). Photographs have been suggested as an alternative to models 33, and the use of photographs, which are easier to obtain, seems to explain this difference. The implications of this different approach to measurement will be explored further in the article analysing the study models.

### The comparability of measures over time

Most outcomes measured in CCUK are consistent and comparable with those measured in CSAG, although there were a couple of exceptions. In the CSAG study, psychosocial adjustment was assessed using a standardized questionnaire of 18 items 19. Over time, these have been modified by the Psychology SIG. The final version of questions used in the study to assess parental/guardian’s perception of the impact of the cleft for their child was agreed by the Psychology SIG and the researchers before the start of data collection. These clinical measures have therefore been modified as the service has evolved. For speech, a validated measure has been developed 17, in contrast to the modified CAPS and a modification of the Eurocleft Speech study for articulation in CSAG 4. Differences between the two approaches are detailed in the speech study. These changes in outcome tools are justified in that they reflect the development of validated tools in the years between the two studies but an unfortunate consequence is that they make the comparisons over time difficult.

### Availability of data and staff from previous surveys

The availability of the original CSAG records is a strength. For example, having photographs from both CSAG and Cleft Care UK study allowed us to analyse them in the same standardized way using improved technology. This allowed direct comparison between the two studies. One of the original CSAG study model assessors and two of the CSAG speech researchers have been involved in the CCUK study which has helped to ensure consistency in the approach across the surveys.

### Where to conduct measurements

The response rate for the Health and Lifestyle Questionnaire and that for the Satisfaction with Service Questionnaire were lower than for measures obtained in the audit clinics. Even with further encouragement from the cleft centres and support from CLAPA, we were unable to increase the response rate substantially. Future surveys should consider whether all the survey data could be collected at the audit clinic visit. We were also restricted to limited reminders by the ethics committee. It would be useful to collect empirical evidence on what levels of reminders participants consider reasonable as the ability to send further written reminders and to phone families may have improved the response rate.

### Data quality in audit clinics

We relied on clinicians to collect data for many of the outcomes we were interested in. Data derived and collected from medical records by cleft centres were standardized through visits by the research team and the individual SIGs. This support, as well as the fact that some of the records are part of clinical management, ensured a reasonable consistency across centres. Few of the cleft units had research nurses and many of the local principal investigators had to support projects for the first time.

### Future research using these surveys

Our analysis of these data and comparisons with the previous CSAG report will continue so that we can explore key characteristics of centralized care associated with outcome and the individual and clinical predictors of outcome. We welcome collaborations to maximize the use of these data. We have already set up one further collaboration with this survey – the Cleft Collective project funded by the Healing Foundation is now collecting DNA and other environmental data to complement the rich phenotypic data obtained in the Cleft Care UK study. This project is at an early stage but aims to enrol 3000+ families, and indicates the scale of research project that is possible within the centralized cleft services that now exist in the UK.

## Conclusions

National cross-sectional surveys are feasible in the NHS in selected clinical groups such as children with cleft lip and palate. There are challenges in ensuring comparability between surveys carried out years apart, and there are also cost considerations in obtaining these data on a regular and consistent basis.

## Clinical relevance

The care of children with oro-facial clefting is complex, is multidisciplinary and extends from the antenatal period into adulthood. Fifteen years ago, the United Kingdom undertook a national survey of two groups (5- and 12-year-olds) of children born with non-syndromic unilateral cleft lip and palate. Many aspects of care and a number of outcomes were poor, and as a consequence, services were centralized. The present United Kingdom-wide study has examined care and outcomes in 5-year-old children, born between 1 April 2005 and 31 March 2007 with non-syndromic unilateral cleft lip and palate, who were treated by these largely centralized services.

## Supporting Information

**Appendix S1. List of local study PIs, Co-ordinators and staff who contributed to the collection of data for the CCUK project.**
