# Supplementary material for: A cross-sectional survey of 5-year-old children with non-syndromic unilateral cleft lip and palate: the Cleft Care UK study. Part 1: background and methodology
Source: Orthod Craniofac Res. 2015 Nov 16;18(Suppl 2):1–13. doi: 10.1111/ocr.12104 (PMC4670715; doi:10.1111/ocr.12104)
Supplement: Supplementary file 1 [file ocr0018-0001-sd1.docx]

**Appendix**. List of local study PIs, Co-ordinators and staff who contributed to the collection of data for the CCUK project.

| **Cleft Site** | **PI** | **Coordinator** | **Other** |
| --- | --- | --- | --- |
| Addenbrookes Hospital, Cambridge | Per Hall | Sue Burgess |  |
| Birmingham Children’s Hospital | Rona Slator | Alexander Levine | Joy Bond; Victoria Clark; Lars Enocson; Alison Jeremy; Bruce Richard; Rachel Sanders; Imogen underwood |
| Frenchay Hospital Park Road, Bristol | Liz Albery | Richard Willerton |  |
| Great Ormond Street Hospital, London | Loshan Kangesu | Laura Sennett | Lauren Baillie; Raouf Chorbachi; James Green; Norman Hay; Anne Mayne; Natalie Pancewicz; Marie Pinkstone; Brijesh Patel; Debbie Sell; Jo Shearer; John Volcano; Karen Wilson |
| Guys & St Thomas Hospital, London | Alex Cash | Peggy Mo | Duncan Atherton; Hannah Cook; Carole Evans; Zoe Jordan; Kate Le Merechal; Sue Mildinhall; Louise Mills; Lucy Partridge; Lucy Smith; Emma Southby |
| John Radcliffe Hospital, Oxford | Stephen Robinson | Steven Berry |  |
| Leeds General Infirmary | Alistair Smyth | Heather Jamieson |  |
| Morriston Hospital, Swansea | Adrian Sugar | Andrea Thomas |  |
| Nottingham University Hospitals | John Rowson | Vicky Nightingale | Claire Benton; Lorraine Britton; Tracey Cooper; Karine Latter; John Rowson |
| Royal Aberdeen Children’s Hospital | Felicity Mehendele |  |  |
| Royal Hospital for Sick Children, Glasgow | Toby Gilgrass | Elaine Simpson |  |
| Royal Hospital for Sick Children, Edinburgh | Felicity Mehendele |  | Gillian Cairns; Margaret Davie; Jacqueline DeLange; Kristina Doran; Órla Duncan; John Hammond; Dawn Lamerton; Paton; Vidya Srinivasan; Steve Stanton; Anna Wilkinson |
| Royal Liverpool Children’s Hospital | Joyce Russell | Helen McCormick |  |
| Royal Manchester Children’s Hospital | Joyce Russell | Claire Richardson |  |
| Royal Victoria Infirmary, Newcastle | Peter Hodgkinson | Paula Spence |  |
| Salisbury District Hospital | Stephen Robinson | Mary Ann Brewer |  |
| St Andrew’s Centre, Broomfield Hospital, Chelmsford | Loshan Kangesu | Karen Wilson |  |
| The Royal Victoria Hospital, Belfast | Chris Hill | Pamela Foster |  |
